# A Case of Late Presentation of Pancreatic Divisum in a Patient with Recurrent Pancreatitis

**DOI:** 10.1155/2020/3437465

**Published:** 2020-07-14

**Authors:** Taranika Sarkar, Sophia Jagroop

**Affiliations:** Jamaica Hospital Medical Center, Internal Medicine, Richmond Hill, NewYork, NY 11418, USA

## Abstract

Pancreatic Divisum (PD) is the most common congenital variation of pancreatic duct anatomy, arising when embryological ventral and dorsal endodermal buds fail to fuse (“classic” PD) or only fuse partially (“incomplete” PD). Most patients with PD are asymptomatic, but a subgroup of patients can present with recurrent bouts of pancreatitis. While alcohol and gallstones are the common causes of acquired pancreatitis, PD is a congenital cause of pancreatitis. It is usually suspected in younger individuals with recurrent pancreatitis who also have a family history. Here, we present a rare case of PD in an older individual who presented with recurrent pancreatitis. He underwent cholecystectomy for suspected gallstone pancreatitis but continued to have episodes of pancreatitis. He had a history of alcohol abuse but denied use in the last one year. PD was detected later as the cause. Recurrent pancreatitis led to the development of a pseudocyst and pancreaticopleural fistula (PPF). Medical management improved the pseudocyst and PPF.

## 1. Introduction

Pancreatic Divisum (PD) is a rare cause of recurrent pancreatitis. Recurrent pancreatitis can result in a clinically significant disability due to chronic abdominal pain, pancreatic insufficiency, pseudocysts, and pancreatic mucinous neoplasms [1]. The abnormal fusion causes abnormal drainage of majority of the pancreatic juice into the minor papilla. A stenosis of the accessory papilla of Santorini can be coexistent. Sometimes there can be ampullary stenosis due to localized ductal ectasia. This leads to high ductal pressure during active secretion, ultimately causing ductal pain [2–5]. Previous studies have shown that low-grade intraductal hypertension makes the pancreas more prone to injury from alcohol, trauma, and drugs [6]. Here, we present a case of recurrent pancreatitis in a middle-aged individual which turned out to be pancreatic divisum. The case highlights the complications of the recurrent pancreatitis when a cause cannot be identified. Also, we present a rare association of PD and pancreaticopleural fistula (PPF) never mentioned before.

## 2. Case Summary

A 52-year-old male with a history of pancreatitis presented with abdominal pain. In his previous admission (3 years ago), ultrasound was significant for cholelithiasis, but there was no common bile dilatation (5 mm) and no cholecystitis. Amylase and lipase were 471 U and 3145 U, respectively. MRCP was negative for CBD dilatation and gallstones. The patient had a laparoscopic cholecystectomy.

**Figure 1:**
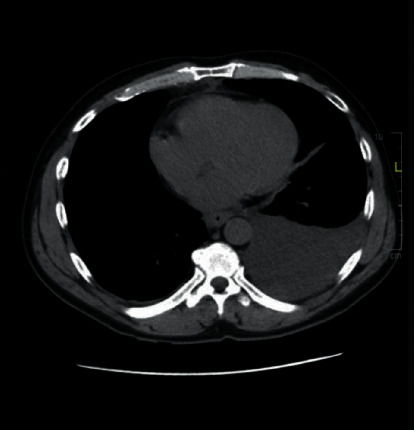
CT scan image of the patient showing pleural effusion on the left.

The patient had a past history of alcohol use, but since few years, he restricted to 3 glasses per week, and in the last 1 year, he had not taken alcohol at all. The patient had 10/10 abdominal pain radiating to the back. Examination was significant for decreased breath sounds bilaterally. Significant tenderness was felt on the left upper quadrant with guarding and rigidity without rebound tenderness. CXR was consistent with left pleural effusion. Amylase and lipase were 320 U and 639 U, respectively. Hemoglobin was 16.8 g/dL, and BUN/creatinine was 11/0.4. The lipid panel was negative for hypertriglyceridemia. CT scan (Figure 1) was consistent with a moderate lateral pleural effusion on the left and tiny on the right.

The patient underwent diagnostic and therapeutic thoracentesis. The fluid was hemorrhagic in appearance. The patient's hematocrit remained stable. Fluid analysis showed an amylase of 12,798, LD of 1218, glucose of 86, protein of 4.4, and albumin of 2.1. It was negative for acid fast bacilli and malignant cells. The tests were suggestive of pancreaticopleural fistula.

**Figure 2:**
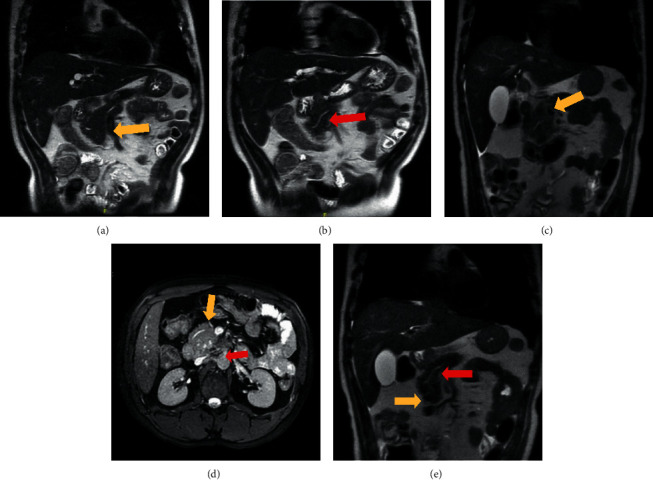
MRCP of the patient showing the accessory duct of Santorini suggestive of pancreatic divisum (yellow arrows show the accessory duct of Santorini, and red arrows show the main pancreatic duct).

The patient underwent MRCP which showed intrahepatic and extrahepatic biliary duct dilatation. The common hepatic duct was 1.5 cm in diameter, and the common bile duct (CBD) measured up to 1.1 cm in diameter. The distal portion of the common bile duct had abruptly changed to 2 mm in diameter. The diameter of the pancreatic duct was normal. There was left-sided pleural effusion and pancreaticopleural fistula. Due to high suspicion of ampullary mass/stricture and stones, a CT scan with IV contrast and pancreatic protocol was performed. It showed a prominent pancreas with ill-defined low attenuation, suspicious of mass. IgG4 antibodies were negative. The patient was planned for an EUS-guided biopsy once his pancreatitis resolved. His PPF and left pleural effusion were monitored and treated with octreotide. In case symptoms worsened, chest tube placement was recommended. However, the patient's condition improved, and he was discharged for an outpatient EUS (endoscopy-guided ultrasound with biopsy). He developed another episode of abdominal pain. Amylase and lipase were 320 and 639, respectively. CT scan showed a pancreas suggestive of an episode of acute pancreatitis. Accessory pancreatic duct of Santorini was identified, which was suggestive of pancreas divisum (Figure 2). There were new bilateral multiple fluid collections suggestive of pseudocysts. Dilation of intrahepatic and extrahepatic of the hepatic duct was stable as the previous CT scan.

The patient was treated for an acute episode of pancreatitis and discharged on a low-fat diet and pancreatic enzyme supplement. Alcohol-induced pancreatitis was unlikely as the patient had <50 g of alcohol per day in the last five years and complete abstinence in the last one year. Pancreatic divisum is associated with pancreatic intraepithelial neoplasia (PIN) due to duct abnormality, and hence, it was essential to follow-up on the ill-attenuated mass. The patient was planned for EUS with biopsy once stable. Two months later, after resolution of pain with no further episodes of pancreatitis, he underwent EUS. It showed a CBD of 4 mm diameter, hyperechoic walls of the pancreatic duct in the head, body, and tail, and parenchyma with hyperechoic foci and lobularity, suggestive of chronic pancreatitis. There were no gall stones. The low attenuated mass was actually a 35 mm × 22 mm sized pseudocyst. It showed the pancreatic divisum as well. As the patient was ruled out from alcohol, gallstones, hyperlipidemia, and autoimmune cause, divisum could be concluded as the cause of recurrent pancreatitis. The patient is currently being evaluated for the MRCP secretin stimulation test to see if he remains symptom-free for a month. This will help to assess for pancreatic reserve and whether patient has coexistent minor papilla stenosis and would benefit from sphincterotomy. Due to high association with PIN and intraductal mucinous neoplasm (IPMN) (9%) [1], surveillance EUS and CTAP pancreatic protocol were ordered 6 months later to assess cyst resolution and monitor changes in the pancreas and duct appearance.

## 3. Discussion

Pancreatic Divisum (PD) occurs in approximately 4–14% of the population [7]. PD has geographic variations, where it is found in approximately 4–10% of the Caucasian population and 1-2% of the Asian population.

There are three major types of PD. Type I, or classic pancreatic divisum, is a complete failure of the dorsal and ventral buds to fuse. Type II pancreatic divisum is characterized by the absence of the ventral duct, so the minor papilla drains the entire pancreas and the major papilla drains some of the common bile duct. Finally, type III presents with a small remnant communication between the dorsal duct and ventral duct [7, 8].

PD is usually asymptomatic, so routine radiological diagnosis is usually not encouraged. Unnecessary sphincterotomy or dorsal duct cannulation can lead to pancreatitis and contrast-induced renal failure in the asymptomatic patients. On the other hand, failure in identification will eventually lead to pancreatic failure or lead to neoplasia and pancreaticopleural fistula. Hence, more studies are required to identify the subset which would benefit from diagnosis and treatment. From our patient's case, it can be emphasized that it is crucial to look for other causes of recurrent pancreatitis and send patients for radiological imaging when more common risk factors such as gallstones and alcohol have been ruled out. It is not necessary that hereditary causes such as divisum will present exclusively in a younger age or with a family history of pancreatitis. Early diagnosis can prevent long-term sequelae of recurrent pancreatitis.

Several case reports show complications associated with PD due to coexistent stenosis of either minor papilla or the ampulla, such as cystic dilation of the dorsal pancreatic duct or obstructing pseudocyst of the duct of Santorini. There are also case reports discussing pancreatic mucinous neoplasm and other pancreatic tumors. Patients with PD and those who have pancreatic type of pain and elevation of pancreatic enzymes should be followed up due to the risk of developing pancreatic cancer.

Another rare but serious complication of PD is pancreaticopleural fistulas (PPFs) which has never been reported. PPFs occur in 0.4% of patients with pancreatitis and in 6–14% of patients with pancreatic pseudocysts [9, 10]. When pancreatic secretions leak posteriorly, they track cranially into the pleura forming PPFs. Involvement of the left pleural space is, by far, the most common, accounting for 76% of the cases. Involvement of both pleural spaces is less common and has been reported in 14% of patients with pancreaticopleural fistulas. PPFs are suspected when pancreatitis present with large hemothorax and high amylase content in pleural fluid analysis. They may present as predominant chest symptoms and recurrent pleural effusion. Due to the stimulation of the pleura by pancreatic exocrine secretions, PPFs should always be drained to provide symptomatic relief and avoid progression of inflammation. After thoracocentesis, somatostatin analogue such as octreotide can be used to decrease pancreatic secretions. If symptoms do not improve, ERCP-guided stent placement is performed for diversion.

Several diagnostic modalities have been developed over the years with different sensitivities and specificities to diagnose PD. ERCP is considered the gold standard for diagnosis. Modern noninvasive imaging has decreased the need of ERCP from diagnosis to therapeutic benefit. MRCP with secretin stimulation has an added advantage of assessing the exocrine pancreatic reserve and the associated minor papilla stenosis.

Endoscopic sphincterotomy of the minor papilla is considered superior to the surgical approach as it is less invasive [11, 12]. Our patient was being planned for the EUS secretin stimulation test to assess minor papilla stenosis and whether he would benefit from sphincterotomy.

We, thus, emphasize high index of suspicion of PD as one of the causes of recurrent pancreatitis even in the middle-aged population.
